# Testing the Efficacy of a 1-Day Police Decision-Making and Autonomic Modulation Intervention: A Quasi-Random Pragmatic Controlled Trial

**DOI:** 10.3389/fpsyg.2021.719046

**Published:** 2021-08-11

**Authors:** Paula Maria Di Nota, Joseph Arpaia, Evelyn Carol Boychuk, Peter I. Collins, Judith Pizarro Andersen

**Affiliations:** ^1^Health Adaptation Research on Trauma Lab, Department of Psychology, University of Toronto, Mississauga, ON, Canada; ^2^Department of Counseling Psychology and Human Services, University of Oregon, Eugene, ON, United States; ^3^Department of Psychiatry, Faculty of Medicine, University of Toronto, Toronto, ON, Canada

**Keywords:** police education, use of force, police training, occupational stress, stress management, autonomic arousal, heart rate variability, biofeedback

## Abstract

Contemporary discourse has identified several urgent priorities concerning police training and education, including: (a) empirically testing and validating the effectiveness of current programming in reducing lethal force decision-making errors; (b) integrating evidence-based content and pedagogical approaches into police curriculum; and (c) understanding the breadth and length of programming necessary to ensure learning and transfer of skills to operational field settings. Widespread calls to identify effective and actionable training programs have been met with numerous research studies, systematic reviews, and policy recommendations that reveal the need to train officers’ internal physiological awareness, which is foundational in shaping cognitive decision-making, emotion regulation, and behavior under stressful conditions. Several investigations have shown improvements to both lethal force errors and physiological recovery following a multi-day autonomic modulation (AM) intervention. Immediate and sustained training gains are observed following repeated practice with clinically validated protocols integrated into training scenarios. Despite evidence-based support for AM in addressing the aforementioned priorities, police organizations are faced with limited time and funding for training and education. The goal of the current quasi-random pragmatic controlled trial was to evaluate the effectiveness of a modified 1-day version of an established AM intervention. A sample of active-duty police officers were quasi-randomly assigned to an AM intervention (*n* = 82) or waitlist control group (*n* = 105). Lethal force errors and objective measures of autonomic arousal and recovery were measured during reality-based scenarios pre- and post-training and at 12-month follow-up. In contrast to previous investigations of longer AM intervention protocols, no significant training-related improvements to behavioral or physiological outcomes were found immediately post-intervention or at follow-up. The current results suggest that single-day training is insufficient to learn the physiological awareness and regulation skills necessary to perform effectively during lethal force encounters, as demonstrated by a lack of immediate or sustained training effects. Practical considerations, such as resource allocation, that may undermine the effectiveness of implementing evidence-based police training are discussed.

## Introduction

In response to numerous high-profile cases of police shootings and civilian deaths, there is mounting demand from both police and public stakeholders to identify effective occupational training programs that reduce lethal force errors (Iacobucci, 2014; Dubé, 2016; Huey, 2018; CCJ, 2021). An additional consequence of lethal force errors includes negative psychological and physical health outcomes among police, which are compounded by repeated and long-term exposure to other traumatic work-related exposures (Violanti et al., 2006; Carleton et al., 2018).

Trauma-exposed occupations including police and other emergency first responders are at heightened risk for developing post-traumatic stress injuries (PTSIs) as a result of their work, including heightened symptoms of depression, anxiety, stress, and burnout (Carleton et al., 2018). Many organizational interventions aim to build resilience, which is a broadly defined concept that refers to an individual’s ability to psychologically recover and/or maintain stable mental health following a potentially traumatic event or prolonged period of chronic stress (Bonanno, 2005; APA, 2012). For the purpose of the current study, we operationalize resilience as the ability to modify autonomic nervous system (ANS) responses to stress and recover from the physiological responses induced by acute stressors, such as increased modulation of cardiorespiratory responses (i.e., heart and breathing rates) and release of corticosteroids (i.e., cortisol), which have been shown to degrade officers’ cognitive and motor skills during high-threat encounters (Anderson et al., 2019; Di Nota and Huhta, 2019).

Numerous types of organizational training programs and interventions have been developed with the goal of reducing the impact of occupational stressors on operational performance and psychological functioning. However, empirical evaluations of these programs reveal limited effectiveness and significant practical challenges to implementing and evaluating training programs within organizational contexts (Beshai and Carleton, 2016; Di Nota and Huhta, 2019; Anderson et al., 2020; Di Nota et al., 2021). One effective approach that promotes both performance and health includes training police officers’ awareness and adaptive management of internal physiological responses to stress (Bennell et al., 2021). In addition to psychoeducational modules that educate officers about stress physiology and adaptive regulation techniques (Arnetz et al., 2009, 2013), autonomic modulation (AM) using biofeedback during working hours or stressful critical incident scenarios have been particularly effective in improving lethal force errors as well as measures of physical and mental health (McCraty et al., 2009; McCraty and Atkinson, 2012; Andersen et al., 2015, 2018; Andersen and Gustafsberg, 2016; Ramey et al., 2016). In spite of these evidence-based findings, police organizations require significant resources to both deliver and empirically evaluate the long-term effectiveness of AM interventions. Building on the extant literature and balancing practical limitations with experimental rigor, the current study aims to investigate the effectiveness of an abbreviated 1-day version of a previously validated 4-day AM and performance intervention within an operating police agency. By utilizing a pragmatic approach, the current study provides insights into the minimum training duration required to observe significant improvements (both immediate and sustained) in lethal force decision-making and AM, both of which greatly impact the health and safety of the public and police.

Several researchers have capitalized on the scientifically established mechanisms underlying AM (see section “Physiological Mechanisms Underlying the AM Intervention” for details) for the purpose of improving resilience and performance among police officers. Visualizing objective physiological measures like heart rate (HR) and heart rate variability (HRV) in real time is known as biofeedback (HRVBF) and was first employed in organizational training by McCraty et al. (2009). Following a 2-day intervention, these researchers found significant improvements in physiological (i.e., cholesterol, blood pressure, HR), psychological (i.e., positive outlook, self-reported distress), and self-reported operational outcomes (i.e., productivity, motivation) among correctional officers. In a follow-up study, McCraty and Atkinson (2012) delivered a 12-h HRVBF intervention to police officers and found similar post-training improvements to self-reported work performance, coping, depression, family relationships, and interpersonal skills. A subset of officers participated in critical incident scenarios to evaluate the effectiveness of the HRVBF intervention on improving AM and performance during simulated high-threat operational contexts. Based on one post-training scenario, the training group (*n* = 12) showed marginally improved performance, significantly greater increases in HR, and no differences in recovery time (i.e., time to return to average resting HR) 7 weeks following the HRVBF intervention.

In order to maximize learning at a neurophysiological level that is robust to the interfering effects of stress, skills training needs to be delivered in the same manner in which it will be used in the field (Di Nota and Huhta, 2019). Accordingly, Ramey et al. (2016) delivered an extended training protocol that involved two 2-h educational HRVBF sessions to police followed by 3 months of recommended skills practice both in the field and at home before and after stressful events. Researchers found that younger participants showed greater post-training improvements to psychological and physiological (C-reactive protein) measures of stress and cardiovascular disease risk. However, the study failed to report compliance to the 3-month recommended HRVBF practice and also suffered from significant loss of physiological data (68%). While these investigations reveal promising post-training improvements to police autonomic regulation and performance, their findings are highly dependent on adherence to, and duration and fidelity of, HRVBF training protocols.

Building on prior literature, Andersen et al. (2015) developed a 4-day immersive AM intervention called the International Performance and Efficiency Program (iPREP). By integrating HRVBF and metacognitive coping skills training directly into reality-based use of force (UOF) scenarios, iPREP is designed to maximize learning, retention, and application of relevant skills in operational contexts where officers are expected to perform effectively under acute stress. Based on pedagogical insights from police and military populations (Driskell and Johnston, 1998), Andersen et al. (2015) first present psychoeducational modules related to AM and resilience promotion. Next, adaptive metacognitive and breathing skills are conditioned by utilizing HRVBF during increasingly intense and stressful critical incident scenarios that are representative of what officers would experience in the field (Staller and Zaiser, 2015). Initial findings demonstrate that the AM intervention was effective among a sample of special forces tactical police officers (i.e., SWAT) and significantly modulated autonomic arousal post-training (Andersen et al., 2015). Subsequent investigations have established significant reductions in police lethal force errors and improved situational awareness and physiological indicators of health both immediately post-iPREP training and sustained for up to 18 months (Andersen and Gustafsberg, 2016; Andersen et al., 2018).

Despite the promising results of the AM studies summarized above, researchers and applied practitioners have highlighted significant practical barriers to consistently delivering and empirically evaluating the effectiveness of training interventions within an operating police agency (Scantlebury et al., 2017; Di Nota and Huhta, 2019; Andersen and Collins, 2020; Anderson et al., 2020). Finite agency resources including time, money, and available qualified personnel (i.e., trainers and participants) also limit the extent to which additional (i.e., non-mandatory) training such as AM and resilience promotion can be delivered to officers. Furthermore, significant resources, partnerships, and coordination with external research teams are needed to empirically validate AM interventions that balance experimental control (i.e., random assignment of participants, intervention and study duration, follow-up evaluations) with operational demands (i.e., shiftwork and staffing).

Addressing the above priorities motivated the goal of the current study: to test the effectiveness of an abbreviated 1-day (i.e., 10 h) version of an established 4-day AM intervention (Andersen et al., 2015, 2016, 2018). Utilizing a quasi-random pragmatic controlled trial design, the current investigation leveraged organizational challenges with scientific rigor to test the effectiveness of a more time- and cost-effective AM intervention protocol. Based on the reviewed multidisciplinary literature on stress physiology, learning, and organizational training interventions (see also section “1-Day AM Intervention Protocol”), we hypothesized that reductions in lethal force errors and improvements in autonomic functioning would be far less pronounced than those following longer intervention durations. The insights gleaned from the current study aim to provide important insights on the necessary training duration required to observe significant improvements in lethal force decision-making and stress modulation following an AM intervention tailored to the occupational demands of the police agency.

## Materials and Methods

### Participants

A total of 187 (31 female) active-duty frontline police officers volunteered for the current study. Demographic information was voluntarily supplied by a sub-sample of officers and is summarized in Table 1. The total pool of active officers at the large urban police service in Ontario, Canada was approximately 750. Study inclusion criteria were any frontline officer who had completed all basic training, were not being supervised by a “coach” officer and were deemed as “fit for duty” according to the service’s standards on the days that researchers attended the service to recruit study participants (see section “Procedure” below). Exclusion criteria were un-sworn police service workers (i.e., civilian staff), officers who were on medical leave or vacation at the time of study recruitment and evaluation, and officers deemed unfit for duty. The police service did not provide an estimate of the number of individuals that met exclusion criteria from the total pool.

**TABLE 1:** Demographic summary.

	Experimental group		Control group	
	Pre- and post-intervention	12-month follow-up	Pre-intervention	12-month follow-up
*n* (Female)	82 (12)	38 (7)	105 (19)	27 (1)
*M* age (SD)	33.3 (6.16)^a^	32.3 (5.67)^c^	34.3 (6.55)^e^	33.6 (6.50)^g^
*M* years of service (SD)	7.48 (5.32)^b^	7.2 (5.75)^d^	8.2 (5.42)^f^	8.0 (5.22)^h^

*^*a*^Data from *n* = 104.*

*^*b*^Data from *n* = 157.*

*^*c*^Data from *n* = 32.*

*^*d*^Data from *n* = 34.*

*^*e*^Data from *n* = 51.*

*^*f*^Data from *n* = 84.*

*^*g*^Data from *n* = 34.*

*^*h*^Data from *n* = 34.*

Considering recommendations regarding pragmatic trials (Patsopoulos, 2011) and the operational demands of the current police agency, allocation of eligible participants to the experimental and control groups was quasi-random such that participation in the current study and AM intervention was secondary to reporting for occupational duties and scheduling. Officers that could return for a full day of AM intervention training within 2 weeks of the baseline evaluation were assigned to the experimental group (*n* = 82), and those officers that could not participate in an additional training day within the 2-week time frame (i.e., due to shift work requirements or vacation schedules) were assigned to the control group (*n* = 105). The control group in this study was an “intent to treat” group in which the intervention materials were provided to police administrators and promised to be delivered to control participants by the organization once the study was complete.

**FIGURE 1:**
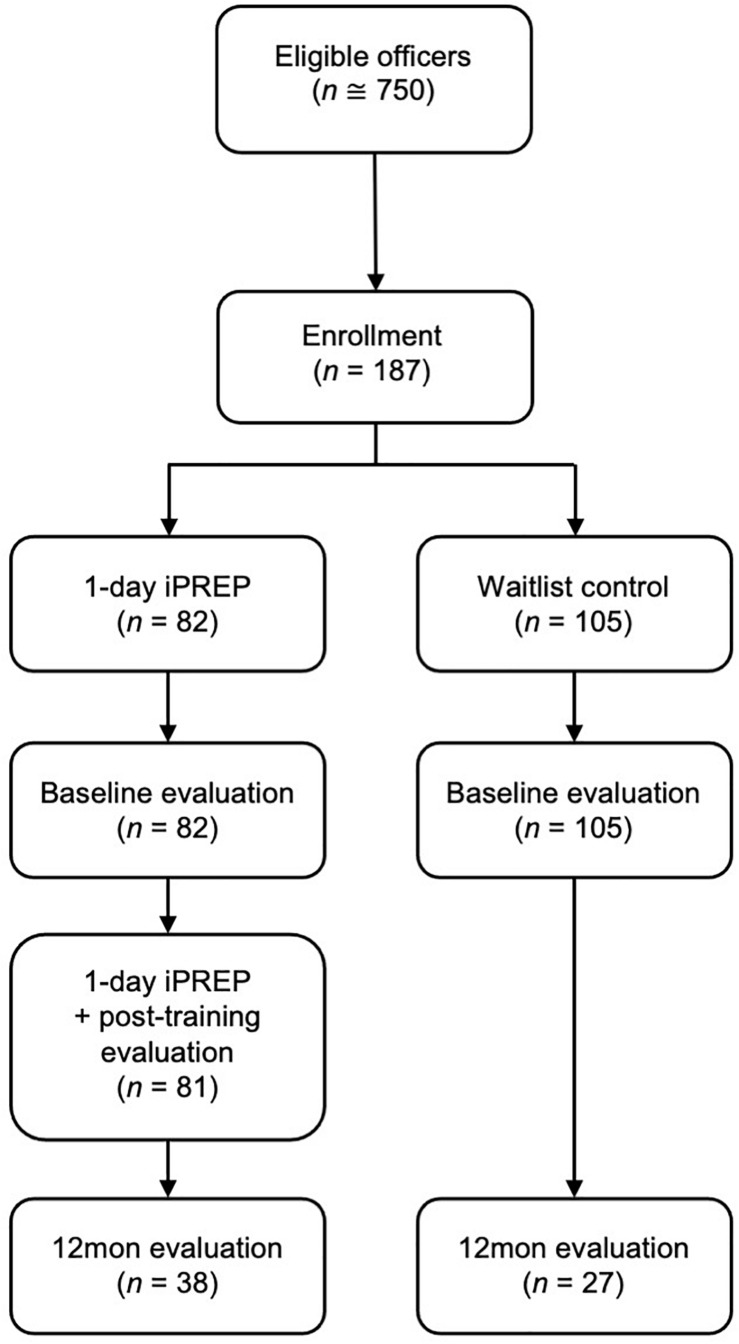
Consort diagram.

Of the original 187 participants, 65 (35%) returned for the follow-up evaluation completed 12-months following the last AM intervention session. No significant differences in age, years of service, or outcome variables (lethal force decision-making, physiology) were observed between participants who returned for follow-up and those lost to attrition (*p*s > 0.10). Given that shift work schedules and planned absences are assigned months and sometimes even a year in advance, we were unable to forecast the level of participant attrition at 12-month follow-up. In order to attend the follow-up evaluation, officers had to be on a day shift and obtain permission from their shift supervisor to take the time to attend. As the police service and researchers were unable to pay officers for overtime to attend the follow-up evaluation, officers were not incentivized to attend on personal time. Flow of participants through the study design are shown in Figure 1.

All participant data was de-identified and is securely stored on password-protected internal servers hosted by the university and accessible only by approved members of the research team. No research data was shared with or stored at the police agency from which participants were recruited, and all police evaluators were blinded to participant group allocation. All research procedures were approved by the University of Toronto Research Ethics Board, and all subjects gave consent in writing prior to participating in the study in line with the guidelines of the Declaration of Helsinki. All de-identified data, materials, and methods can be made available upon request to the corresponding author.

### Procedure

#### Baseline/Pre-intervention Evaluation

Researchers were allowed to recruit participants for the baseline evaluation and 1-day AM intervention during the agency’s annual UOF/firearms recertification days, which included scenario-based evaluations and firearms testing, between 2017 and 2018. Members of the research team attended morning roll call and described the study aims and requirements of the 1-day AM intervention. Those officers who volunteered to participate in the study would be observed during their recertification scenarios by members of the research team to collect their behavioral data (i.e., lethal force decision-making) in addition to physiological HR data for research purposes. All potential volunteers were informed that participation in the research study was independent from their agency evaluation. Upon volunteering, participants were taken to another room, given a verbal explanation of the consent form, then given time to read the consent form and sign it. Once participants provided their written informed consent they were fitted with medical-grade HR monitors (BodyGuard 2, FirstBeat Technologies Ltd., Jyväskylä, Finland). Next, participants were assigned to the experimental or control group according to the criteria and procedures described above. Prior to returning to the larger group to complete their annual recertification assessments, resting HR was obtained while officers were in a seated position for 5 min (see section “Measures”).

As researchers were not permitted to attend firearms assessment, lethal force decision-making was observed during reality-based scenarios that were designed by UOF instructors, who were not part of the research team, to evaluate officers’ UOF decision-making and situational awareness skills. Reality-based scenarios involve actors, staged environments, and props to increase the ecological validity of simulated encounters. Pre-recorded video scenarios were used at pre-intervention and 12-month follow-up evaluations (three at each time point) because the partnering police service had integrated them into their annual recertification evaluation program as a more resource-efficient way of offering additional training and evaluation scenarios. For all scenarios, participants were outfitted with training versions of all their standard issue police gear where all projectiles were removed [e.g., training pistol, inert oleoresin capsicum (OC) spray, training baton, and training conductive energy weapon (CEW)]. Consistent with the police service’s recertification procedures, scenarios were completed in pairs. To ensure that members of the research team could adequately record the start and stop time of the scenarios (i.e., to match to HR data) and behavioral outcomes (i.e., whether or not a lethal force error was made), only one officer in each pair was a study participant. Police instructors were blinded to which officer was a study participant and to their group assignment.

All participants completed two live scenarios and three video scenarios during the pre-intervention evaluation that involved typical encounters attended by officers at that service (see Reality-Based Scenario Descriptions). Each scenario required either a “shoot” decision (i.e., using lethal force when situational requirements had been met) or “no-shoot” decision (i.e., not using lethal force and instead employing verbal and/or non-lethal force technique according to situational requirements). Scenarios were designed by certified police UOF instructors in accordance with provincial recertification standards and were not designed or scored by the researchers.

Upon completion of each scenario, the facilitating instructor scored officers by filling out an evaluation sheet, as per standard assessment protocols, and provided them with verbal feedback on their performance. For the purpose of this study, instructor-rated errors in participant’s use of lethal force was obtained (i.e., shooting when they were not supposed to, or failing to shoot when required to). At the end of the scenario training block, officers’ HR monitors were removed and returned to the research team prior to returning to their regular duties (i.e., continued assessment or work duties).

#### Follow-up Evaluation

Participants in the experimental and control groups were invited to participate in a 12-month follow-up evaluation day that was not part of their agency’s annual recertification procedures, but that the officers were paid to attend if permitted as part of their regular duties. A total of *n* = 38 (46% return rate) participants from the experimental group and *n* = 27 (26% return rate) participants from the control group attended 12-month follow-up evaluations. Similar to pre-intervention procedures, officers were fitted with training equipment and HR monitors prior to providing 5 min of seated resting HR data and completing scenarios, which were facilitated and evaluated by certified UOF instructors. All participants completed two live and three video scenarios that were designed by UOF instructors to reflect a similar level of complexity and number of lethal force decision-making opportunities as the pre-intervention scenarios (see Reality-Based Scenario Descriptions).

### 1-Day AM Intervention Protocol

The current 1-day AM intervention was a modified version of a previously established 4-day AM protocol (Andersen et al., 2015, 2016, 2018). The components of the modified protocol are as follows:

• Psychoeducation: The intervention began with approximately 60 min of psychoeducational classroom instruction on stress physiology, and its impacts on decision-making and performance. Related topics included the link between the brain, heart and lung function, chronic stress, and how situational awareness can be affected during stress. Content was gleaned from recent research articles on stress, health, and performance cited in the introduction of this paper and in previous publications (see Andersen and Gustafsberg, 2016).
• HRVBF: Given the shortened program length, officers were introduced to HRVBF using the “Inner Balance” application in the “Coherence Advantage” app (HeartMath, Inc.). This HRVBF program was the only commercially available app at the time of the study that could provide an HRVBF training protocol in a condensed time frame. Participants were given a short (∼30 min) opportunity to learn the HRVBF equipment and ‘‘Coherence’’ techniques. As described by HeartMath, coherence is a state in which the SNS and parasympathetic nervous system (PNS) are synchronized, producing an internal physiological state that promotes PNS dominance.^1^ According to the HeartMath instructions, coherence is achieved in the following way: the app provides a screen displaying a circle that increases and decreases in size corresponding to the time it takes to inhale and exhale at a pace of 5 breaths per minute. A pulse oximeter clipped to the ear (Inner Balance Coherence Sensor, HeartMath, Inc.) communicates with the app and provides a biofeedback signal to the participant indicating how “successful” they are at achieving a coherent state by synchronizing the SNS and PNS through the prescribed breathing pace. If the person achieves a coherent (i.e., synchronized) state, they are “rewarded” with a green light on the app interface. In addition to the 30-min orientation and practice, participants were instructed to engage in the HRVBF techniques immediately before and after each scenario and in downtime between scenarios (see next section).
• Reality-based training scenarios: Scenarios were designed and facilitated/scored by two separate groups of certified UOF instructors that were independent of the research team in order to ameliorate any bias (i.e., in scenario design and outcomes). Following the HRVBF training, officers participated in seven live reality-based scenarios increasing in complexity and stress over the course of the day. Scenarios required officers to perform all relevant skills related to UOF encounters (i.e., de-escalation, tactical skills, verbal and physical communication, situational awareness). Participants were instructed to practice the HRVBF techniques immediately before and after each scenario in order to modify physiological responses to acute stress, as well as to increase the level of both PNS and SNS activation during scenarios and promote faster post-scenario recovery through PNS modulation. A member of the research team observed each participant as they completed the HRVBF techniques.

During the time between scenarios, participants engaged in several guided exercises in order to practice some of the theoretical topics introduced in the psychoeducational module, including:

• Component skills training to combat cognitive deficits: Police UOF training can often place officers directly into high-stress scenarios (i.e., “stress inoculation”) without the opportunity to gradually learn de-escalation and UOF decision-making skills in a calm state and non-complex scenario. Evidence from adult learning theory and cognitive psychology clearly suggests that individual discrete or “component” knowledge and skills must first be learned before combining them into complex combinations that can be effectively employed in stressful contexts (Holmes and Collins, 2001; Di Nota and Huhta, 2019). Together with increasingly complex reality-based scenarios, expert trainers guided participants through critical and appropriate tactical and UOF responses as situations become increasingly faster and more time pressured.
• Enhancing sensory awareness: Once the officers have used the HRVBF device to determine they are in an adaptive physiological state to learn and perform, they engaged in discussion with the expert trainers on how sensory cues (e.g., lights, sirens) may prime or activate physiological stress responses, especially during high-stress situations (Divine, 2013).
• Visualization and mental rehearsal: Due to time restraints and modifications for the current 1-day protocol, officers were only able to engage in this exercise once. Following one reality-based training scenario, officers were asked to assume a comfortable position and mentally picture performing the previous scenario while maintaining a calm state. Officers were also instructed to visualize their actual performance, and then mentally rehearse optimal performance of the scenario by integrating the instructions and feedback from expert trainers. Mental rehearsal and visualization of optimal performance while in a calm state has been shown to solidify newly learned knowledge and aid in memory retention for new skills (Holmes and Collins, 2001). Mental rehearsal techniques have also been used to successfully improve police officer’s performance and reduce threat perceptions prior to critical incident scenarios (Arnetz et al., 2009, 2013).

The AM training day ended with participants completing the same post-training scenario as participants in the 4-day intervention (Andersen et al., 2018), which was an extended active-shooter scenario that required two no-shoot decisions and one shoot decision.

#### Physiological Mechanisms Underlying the AM Intervention

Described elsewhere in greater detail (LeDoux and Pine, 2016), “fight or flight” responses are triggered by activation of the sympathetic branch (SNS) of the ANS following exposure to an acutely threatening or stressful situation. The SNS operates in coordination with the PNS, which is responsible for promoting restorative and regulatory processes in the body (e.g., immune functioning, growth, and repair). Healthy coordination between the SNS and PNS promotes effective functioning, recovery, and health, while overactivation of the SNS in the absence of PNS activity has been shown to impair perceptual and cognitive processes related to police UOF decision-making (Klinger and Brunson, 2009; Haller et al., 2014; Laborde et al., 2014; Roos et al., 2017).

An objective indicator of the coordination between the SNS and PNS is HRV, which can be measured by several metrics including respiratory sinus arrhythmia (RSA). RSA refers to the variation in the intervals between individual heartbeats as a person inhales and exhales. Specifically, HR increases during inhalation (shortening the time between heart beats) and slows during exhalation (lengthening the time between heart beats) (Lehrer and Gevirtz, 2014). As such, RSA reflects synchrony between the heart and respiratory system and plays a key role in the physiological basis of resilience. Clinical studies indicate that increasing RSA builds physical wellness capacity by strengthening an individual’s long-term physiological reserves, theorized to occur *via* multiple pathways (i.e., baroreflex control, blood pressure regulation, and efficiency of pulmonary gas exchange) (Lehrer and Gevirtz, 2014; Lehrer, 2018). Psychophysiological research reveals a connection between respiration, arousal, and emotional control, such that the manner by which a person breathes sends signals to regions in the brainstem and forebrain that modulate arousal. For example, periods of slow breathing are associated with PNS stimulation, which may underlie the anxiety and stress-reducing effects of RSA (Mather and Thayer, 2018).

### Measures

#### Lethal Force Decision-Making

Lethal force errors resulted from: (1) as the primary officer in each pair, failing to shoot during a shoot scenario (i.e., error of disinhibition); or (2) shooting during a no-shoot scenario (i.e., error of inhibition). Criteria for correct or incorrect performance were defined and evaluated by certified UOF instructors independent from the research team. Officers received a score of 1 for each decision-making error and scores were analyzed as error rates (i.e., dividing the total number of errors at each time point by the total number of decision-making opportunities and converting to a percentage).

#### Autonomic Activation and Recovery

Participants had continuous HR data recorded at a rate of 1 Hz (1 recording/s) using Bodyguard 2 cardiovascular monitors (FirstBeat Technologies Ltd., Jyväskylä, Finland). The monitors were affixed to the torso using adhesive electrode patches and worn under the officer’s clothing and equipment during all evaluations. At the end of each day, de-identified data was uploaded from each monitor to an external server for later offline analyses. Two cardiovascular measures were analyzed in the present study. The first is HR_Index, which is an index of the average peak HR (HR_Max) measured as the 5 s before and after the maximum HR achieved during each critical incident scenario relative to each officer’s own average resting baseline HR (HR_Rest) recorded at the beginning of each evaluation day. HR_Index is computed as [(HR_Max − HR_Rest)/HR_Rest]. HR_Max was equal to or less than average HR_Rest in only *n* = 1 of 316 total cases, resulting in a negative HR_Index value. The second cardiovascular measure is HR recovery time (HR_Recovery), or the time (in seconds) for the officer’s HR_Max to return to HR_Rest, which has been reported as an indicator of vagal (PNS) function (Thayer and Sternberg, 2006). HR_Max and HR_Recovery values were averaged across scenarios to obtain a single value at each time point for each officer and were included in statistical analyses described below.

To calculate the HR values used in this study, research assistants manually recorded the following events in the timestamped HR datasheet for each participant and on each evaluation day: (1) start and end time of 5 min of seated rest prior to any assessments to calculate HR_Rest; (2) start and end times for each live scenario and for the blocks of video scenarios in order to identify the 11-s average maximum HR (HR_Max), which were averaged at each timepoint. HR_Max was calculated by identifying the maximum HR achieved during each scenario (or block) and averaging this peak value with the HR 5 s before and after to compute a more stable value of maximal autonomic arousal and adjust for any single beat errors as in Andersen et al. (2018); (3) recovery time was manually extracted from the data by determining the number of seconds it took from the peak HR within each scenario (or block) for the officer to return to their HR_Rest for that day. If an officer did not return to a value equal or lesser than HR_Rest either before the start of the next scenario or before the end of the datasheet, no value was entered (*n* = 51, equally distributed between experimental and control groups at pre-intervention and 12-month follow-up evaluations) and were excluded from those specific analyses, but remained in the study overall. Of note, ∼27% of officers did not return to resting baseline, indicating no recovery during working hours. Data imputation methods were deemed inappropriate to use for missing values due to officers finishing the study at different times, and some took more or less time between scenarios.

Due to technology failure, three officers had no HR data at pre-intervention evaluation, eight officers are missing HR_Rest values (six pre-intervention, two at follow-up), and two officers (one experimental, one control) are missing all pre-intervention HR_Max values but have HR_Rest values. Outlier analyses reveal no participants had HR_Max values that exceeded three standard deviations from the mean. De-identified raw data used in the current statistical analyses are available upon request from the lead author and will require signing a confidentiality agreement to protect participant identity.

### Statistical Analyses

To determine the number of participants necessary to detect effects, we used G^∗^Power to conduct an *a priori F* test (ANOVA: repeated measures, within-between interaction) power analysis with the following parameters: effect size *f* = 0.25, α = 0.05, power (1 − β) = 0.95, number of groups = 2, number of measurements (repeated-measures) = 2. The power analyses indicated a total sample size of 54 participants. Because our total sample size at each time point (pre-intervention: *n* = 187, 12-month follow-up: *n* = 65) exceeds this minimum, we have sufficient power to detect significant effects.

Normality assumptions were evaluated for all outcomes (lethal force error rates, HR_Index, HR_Recovery) at each time point (pre-intervention, post-training, 12-month follow-up) with Shapiro-Wilk tests. All lethal force error rates, all HR_Recovery, and 12-month HR_Index were not normally distributed (*p*s < 0.05) and were evaluated with non-parametric Mann Whitney *U* tests for between-group analyses (*k* = 2) and Wilcoxon Signed Ranks tests for pairwise within-group analyses. HR_Rest and HR_Max were normally distributed and compared using a paired samples *t*-test to evaluate whether critical incident scenarios elicited significant autonomic arousal relative to rest (see manipulation check).

To adjust for multiple planned comparisons (1 between-groups + 3 within-group time analyses × 3 variables = 12 pairwise analyses), an adjusted Bonferroni-corrected value of *p* = 0.05/12 = 0.004 was used to establish statistical significance. All analyses were conducted in SPSS (Version 27, IBM Corp., Armonk, NY, United States).

## Results

### Lethal Force Decision-Making Errors

**FIGURE 2:**
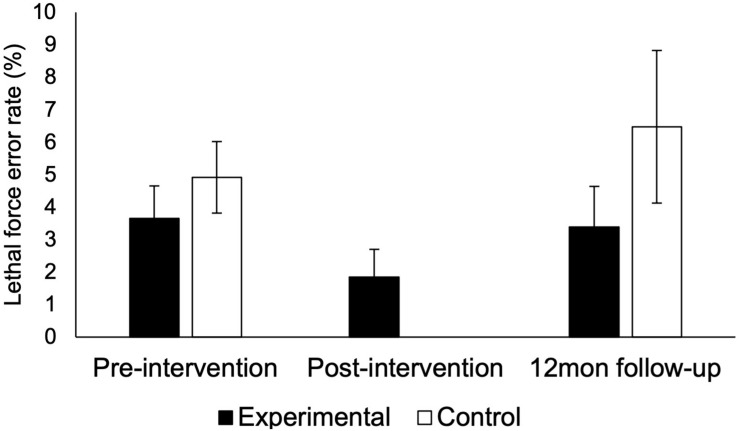
Lethal force decision-making errors. Differences in lethal force error rates between groups and timepoints did not reach statistical significance. Error bars show standard error of the mean.

Overall, very low lethal force error rates were observed for both groups and over time (Figure 2). While error rates decreased from 3.7 to 1.9% immediately following the 1-day AM intervention, these changes were not statistically significant (*z* = −0.746, *p* = 0.46) and returned to 3.4% at 12-month follow-up (*z* = −1.639, *p* = 0.10). Error rates did not significantly differ for the control group at pre-intervention (i.e., baseline) (4.9%) and 12-month follow-up (6.5%, *z* = −1.098, *p* = 0.27). Between-group differences in error rates did not reach statistical significance even at uncorrected levels [pre-intervention: *U*(187) = 4100.5, *p* = 0.41; 12-month follow-up: *U*(65) = 443.5, *p* = 0.22].

### Autonomic Arousal

To ensure reality-based scenarios elicited sufficient autonomic stress arousal, changes between HR_Rest and HR_Max were evaluated at each time point with a manipulation check. Resting HR and HR_Max did not differ significantly between groups at each time point at corrected levels of significance (*p*s > 0.05/4 = 0.013), therefore analyses and HR values reported in Table 2 are averaged across groups. As with the HR_Index analyses, HR_Max was averaged across multiple reality-based scenarios performed during each evaluation timepoint. All scenarios revealed significant HR responses (*p* < 0.000) with large effect sizes according to Cohen’s *d* (Cohen, 1988). Resting HR did not differ across time points [*F*(2, 62) = 0.627, *p* = 0.537], and thus did not confound the HR_Index analysis described below.

**TABLE 2:** Manipulation check: stress responses to critical incident scenarios.

Time	HR_Rest *M* (SD) *n*	HR_Max (during scenario) *M* (SD) *n*	*t*	*df*	*p*	Effect size (*d*)
Pre-intervention	78.79 (12.73) 177	117.53 (17.48) 181	−32.280	175	0.000	2.43
Post-intervention*	80.12 (10.31) 80	139.15 (18.02) 77	−28.295	76	0.000	3.22
12-month evaluation	79.48 (10.21) 62	115.50 (19.42) 64	−19.391	62	0.000	2.44

Heart rate was measured in beats per minute (BPM). Average resting heart rate (HR_Rest) was obtained during 5 min of seated rest at the beginning of each evaluation day. Maximum heart rate (HR_Max) was averaged across multiple lethal force scenarios at each evaluation timepoint except for the post-intervention, which was one prolonged scenario involving three lethal force decisions. Means (*M*), standard deviations (SD), and the number of participants included in each analyses (*n*) are provided for each time point. *Only includes participants in the experimental group that participated in the 1-day AM intervention.

**FIGURE 3:**
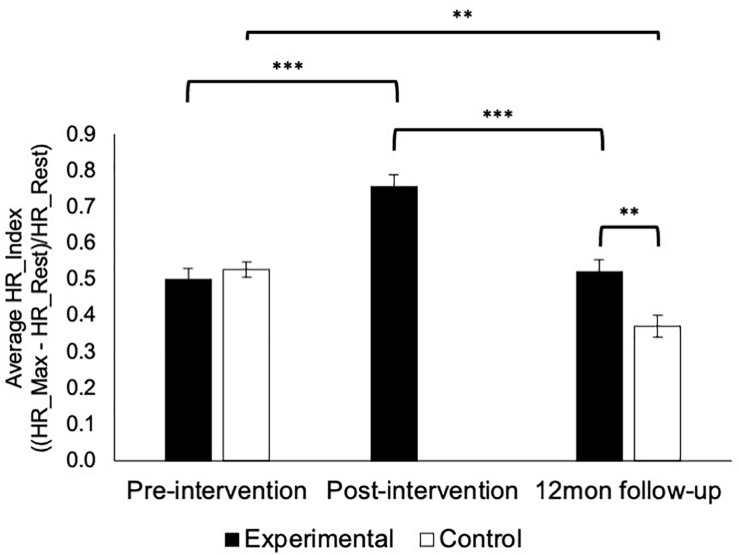
Autonomic activation during critical incident scenarios. HR_Index increased significantly post-training among the experimental group before returning to pre-training levels at 12-month follow-up (*p*s < 0.000). HR_Index also significantly decreased among the control group at 12-month follow up (*p* = 0.002) and was lower than the experimental group (*p* = 0.004). Error bars show standard error of the mean. ^∗∗∗^*p* < 0.001, ^∗∗^*p* < 0.01.

Relative increases in HR as measured by HR_Index did not differ between groups pre-intervention [*U*(176) = 3549.0, *p* = 0.50], but was significantly lower in the control group at 12-month follow-up [*U*(63) = 690.0, *p* = 0.004, Figure 3]. In addition, HR_Index increased significantly post-training for the experimental group (*z* = 5.551, *p* = 0.000) and returned back to pre-intervention levels at 12-month follow-up (*z* = 4.565, *p* = 0.000). HR_Index also significantly decreased between pre-intervention (i.e., baseline) and 12-month follow-up among the controls (*z* = 3.029, *p* = 0.002).

**FIGURE 4:**
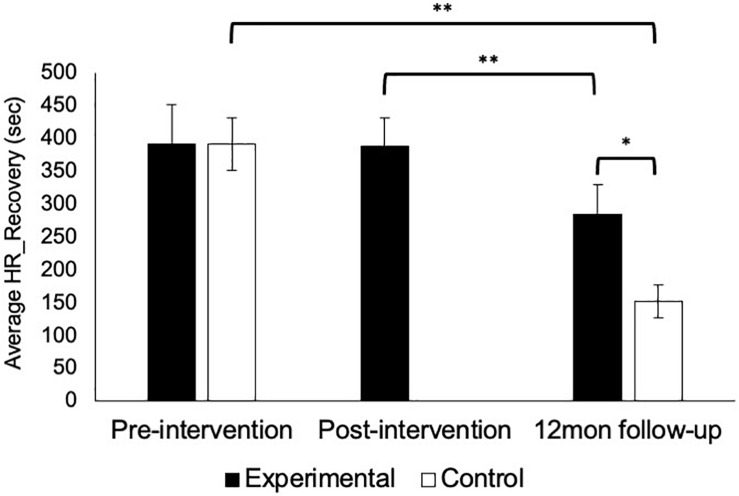
Recovery from peak autonomic activation following critical incident scenarios. HR_Recovery did not significantly change following a 1-day AM intervention, but was faster at 12-month follow-up (*p* = 0.006). Participants in the control group also showed faster HR_Recovery at 12-month follow-up relative to pre-intervention values (*p* = 0.001), which was also faster than the experimental group at uncorrected significance levels (*p* = 0.012). Error bars show standard error of the mean. ^∗∗^*p* < 0.01, ^∗^*p* < 0.05.

HR_Recovery time did not differ between groups at pre-intervention [*U*(127) = 1714.0, *p* = 0.21] but was faster among the control group at 12-month follow-up at uncorrected significance levels [*U*(64) = 678.0, *p* = 0.012, Figure 4]. HR_Recovery was faster among the experimental group from post-intervention to 12-month follow-up at uncorrected significance levels (*z* = 2.763, *p* = 0.006) and significantly faster from pre-intervention to 12-month follow-up among the control group (*z* = 3.285, *p* = 0.001).

## Discussion

The current study sought to investigate the effectiveness of a modified 1-day version of an established AM intervention that has been shown to reduce lethal force errors and improve autonomic functioning (i.e., activation during scenarios and post-scenario recovery from acute stress) (Andersen et al., 2015, 2016, 2018). Using a quasi-random pragmatic controlled trial study design, the current findings reveal no significant differences in lethal force errors between experimental and control groups (Figure 2). Additionally, physiological indicators of autonomic activation (Figure 3) and recovery (Figure 4) were not significantly improved for the experimental group that received the modified 1-day AM intervention, but were higher among the control group. Of note, a substantial number (27%) of officer’s did not recover (i.e., return to resting baseline) during the observation period, indicating a significant need for stress modulation skills. Despite calls for reform to police UOF training and education practices (Iacobucci, 2014; Dubé, 2016; Huey, 2018; Bennell et al., 2021; CCJ, 2021) and growing need for effective occupational health interventions (Weiss, 2019; Anderson et al., 2020; Di Nota et al., 2021), the current findings demonstrate that utilizing abbreviated training to save costs is not effective for rewiring core physiological and cognitive processes aimed at modulating stress responses to reduce UOF errors.

### The Science Behind Police Resilience, Learning, and Performance Under Stress

Motivation to conduct the current study was informed by multiple perspectives, including an urgent need to identify effective training approaches that reduce lethal force errors (Iacobucci, 2014; Dubé, 2016; Huey, 2018; CCJ, 2021). Increased impairment to officer perception, UOF decision-making, and skilled motor behavior have been observed under conditions of increased threat (Klinger and Brunson, 2009; Nieuwenhuys et al., 2009, 2012). Due to the very nature of police work, officers are repeatedly exposed to a variety of potentially psychologically traumatic exposures (PPTEs) (Carleton et al., 2019). Stressful occupational exposures manifest as dysregulated stress physiology observed in both training and active-duty settings as well as in normative diurnal cortisol (Anderson et al., 2002; Andersen et al., 2016; Baldwin et al., 2019; Planche et al., 2019). Long-term exposure to occupational stress increases police officers’ risk for developing PTSIs (Carleton et al., 2018) and suicidal thoughts, plans, and attempts relative to the general population (Di Nota et al., 2020). Accordingly, the current study also addressed an urgent need to identify effective organizational programming that supports resilience and wellness capacity in public safety professionals (Beshai and Carleton, 2016; Weiss, 2019). Existing organizational training programs that aim to build resilience and mitigate the onset of PTSIs following PPTEs showed limited effectiveness, but with promising results for protocols integrating HRVBF including iPREP (Di Nota et al., 2021). A recent paper by expert police researchers and practitioners posits that training police officers to recognize and modify internal physiology during stressful encounters is one of the most urgent training approaches to address lethal force errors and promote physiological resilience (Bennell et al., 2021). AM requires significant rewiring of highly complex and implicit physiological processes (i.e., RSA, HRV) *via* explicit practice with self-awareness (i.e., introspection) and breathing techniques (Lehrer et al., 2013; Arpaia and Andersen, 2019). Research on short (i.e., 4–6 h) police training durations show limited generalizability of complex police arrest and self-defense skills from training to field application (Renden et al., 2015). Together with the current results, these findings call into question the effectiveness of UOF training that lacks sufficient time for officers to learn, practice, and retain these skills under stressful conditions when implicit strategies are favored over explicit ones (Roos et al., 2017; Hine et al., 2018).

### Balancing Organizational Needs and Barriers With Learning Theory

At the time the current study was conceptualized and delivered (2015–2017), there was a strong organizational demand to reduce the duration of police training and skills-based practice based on an increasing number of government-mandated certification and policy initiatives. Subsequently, systematic reviews of 1-day organizational training protocols among first responders have shown limited improvements to resilience that are not sustained (Di Nota et al., 2021).

The current AM intervention is the only organizational resilience promotion program that evaluates and seeks to improve complex cognitive, motor, and physiological regulation skills relevant to operational UOF decision-making (and specifically lethal force) under stress. The current findings confirm that shorter AM training comes at the cost of previously observed short- and long-term gains for longer HRVBF training protocols (Arnetz et al., 2009, 2013; McCraty et al., 2009; Andersen et al., 2015, 2016, 2018).

The existing empirical literature evaluating whether longer police training durations translates to greater gains in officer UOF performance is very limited. A recent study compared the effectiveness of single session, distributed (i.e., “booster” training), and block training approaches for defensive and control tactics in three recruit academies in the United States. Across protocol types that all consisted fewer than 80 min of training for any given skill, immediate post-training gains were lost over long-term follow-up (O’Neill et al., 2019). All of these protocols were representative of the type and duration of training offered at each police agency, which has to balance instructional needs with operational demands (i.e., number of available officers for training or field duty at any given time). Drawing from learning theory, the deliberate practice framework has been suggested as an effective approach to acquiring knowledge in police work (Wolfe et al., 2020). Specifically, deliberate practice requires: (1) instruction and performance on a defined task, (2) immediate feedback, and (3) ample opportunity for repetition to refine performance (Ericsson, 2004). The current findings suggest that 1 day is simply not enough time to engage in effective deliberate learning strategies to promote gains in UOF performance and AM, which have been observed at longer training durations for the same protocol (Andersen et al., 2015, 2016, 2018).

Due to pronounced logistical constraints related to available training time and resources at the police agency where the current study was conducted, each component of the established AM training protocol was shortened or modified, resulting in a dramatically condensed learning exposure. As expressed by other police researchers, Wolfe et al. (2020) state that successful training programs “must be modified to fit the reality of policing operations. It is not possible to have officers engage in hours of deliberate practice like military personnel or competitive athletes. In other words, a successful deliberate practice program must find a way to increase the repetition of exercises […] without unduly burdening the operational demands of a department” (p. 130). Therefore, the current investigation of a modified and abbreviated training intervention was a legitimate and urgent question to test. Consistent with the science of learning and retention (Kang, 2016), a lack of demonstrated benefit following 1 day of exposure to a variety of new concepts is unsurprising.

### Limitations

Contrary to predicted improvements in autonomic regulation in the experimental group, HR_Recovery and HR_Index were higher at 12-month follow-up among the control group. While these unexpected findings are likely due differences in sample size and attrition, they also reflect central study limitations and pragmatic realities of applied police research. One such limitation was the relatively low recruitment rate that precluded larger sample sizes, lack of true random assignment to experimental and control conditions, and which also pronounced the effects of participant attrition. Failing to return for long-term follow-up evaluations undermines researchers’ ability to evaluate potential sustained effectiveness of a training intervention. Although it could not be confirmed, attrition of the current samples can be attributed to several potential factors: a lack of research funding for incentive payments to off-duty participants, lack of interest in the study and/or optional AM intervention, officer turnover (i.e., assignment to different role or precinct), sick leave, vacation, or operational demands (i.e., assignment to night shifts, sudden changes to schedule for required duty on scheduled follow-up days).

Another study limitation includes the biofeedback protocol employed. At the time of the study, we used the Coherence Advantage HRVBF program (HeartMath, Inc.). Established HRVBF protocols and programs that aim to rewire autonomic functioning, including HeartMath, recommend users to engage with the training protocol for *multiple days and sometimes even weeks* at a time (McCraty et al., 2009; McCraty and Atkinson, 2012; Lehrer et al., 2013). In prior studies, the full AM protocol included longer psychoeducational, HRVBF, and active scenario-based practice sessions. Thus, deviating from the established protocol by using the biofeedback technology alone and for a limited time may not be sufficient to offer previously observed benefits in police (Arnetz et al., 2009, 2013; McCraty et al., 2009; Andersen et al., 2015, 2016, 2018). The ambulatory physiological measures used in the current study precluded separating autonomic arousal from physical (i.e., aerobic activity) sources. However, movement was restricted during reality based scenarios given the constrains of the rooms (e.g., no running or even fast walking was possible). Thus, the HR measure is the most robust biomarker as it is not significantly varied in response to minor movements given the low oxygen demands placed on the body while standing or shifting position.

## Implications and Conclusion

The growing number of instances where police officers misapply force due to stress-induced decrements to their perception, judgment, and decision-making demand an empirical evaluation of current police training and education practices.

The current study suggests that: (1) the psychophysiological mechanism (i.e., ANS modulation) that regulates UOF decision-making was not sufficiently trained in a modified 1-day protocol; and (2) there is an urgent need to experimentally examine all short duration UOF training protocols for effectiveness in skill development and generalizability to operational conditions. According to basic scientific evidence, a few hours of practice is simply not enough time to condition adaptive physiological responses and complex lethal force decision-making under stressful and realistic conditions. Effective learning requires initial encoding, as well as deliberate and repeated practice in varied, dynamic, and increasingly stressful contexts.

## Data Availability Statement

The datasets presented in this article are not readily available because demographic information (i.e., sex, age, years of service) may potentially identify research participants. Requests to access the datasets should be directed to JA, judith.andersen@utoronto.ca.

## Ethics Statement

The studies involving human participants were reviewed and approved by University of Toronto Social Sciences, Humanities and Education Research Ethics Board. The patients/participants provided their written informed consent to participate in this study.

## Author Contributions

PD: data analysis and manuscript writing. EB: data collection, organization, and analysis and manuscript preparation. JA: conceptual framework, intervention co-development (HRVBF, cardiovascular physiology), and manuscript writing. PC: study conceptualization, study design, and editing. JPA: conceptual framework, co-development of the intervention (psychophysiology), data collection, and manuscript writing. All authors contributed to the article and approved the submitted version.

## Conflict of Interest

The authors declare that the research was conducted in the absence of any commercial or financial relationships that could be construed as a potential conflict of interest.

## Publisher’s Note

All claims expressed in this article are solely those of the authors and do not necessarily represent those of their affiliated organizations, or those of the publisher, the editors and the reviewers. Any product that may be evaluated in this article, or claim that may be made by its manufacturer, is not guaranteed or endorsed by the publisher.

## Footnotes

1
https://www.heartmath.com/science/
